# Non-Disclosure of Violence among Female Sex Workers: Evidence from a Large Scale Cross-Sectional Survey in India

**DOI:** 10.1371/journal.pone.0098321

**Published:** 2014-05-20

**Authors:** Bidhubhusan Mahapatra, Madhusudana Battala, Akash Porwal, Niranjan Saggurti

**Affiliations:** HIV and AIDS Program, Population Council, New Delhi, Delhi, India; University of Missouri-Kansas City, United States of America

## Abstract

**Objective:**

One of the indicators critical to the success of violence reduction programmes among female sex workers (FSWs) is the pattern of disclosure of violence. This study examines the rate of non-disclosure of violence among FSWs in India by perpetrators of violence and programme exposure.

**Methods:**

Data were drawn from a cross-sectional study conducted among FSWs in 2009 across four states of India: Andhra Pradesh, Karnataka, Maharashtra and Tamil Nadu. The analytical sample included 1341 FSWs who experienced physical violence in past six months. Multilevel logistic regression stratified by state was conducted to examine predictors of non-disclosure.

**Results:**

About 54% of FSWs did not disclose their experience of violence to anyone with considerable variations in the pattern of disclosure across states. Another 36% of FSWs shared the experience with NGO worker/peer. Compared to violence perpetrated by paying partners/stranger, that by non-paying partner were twice more likely to report non-disclosure (53% vs. 68%, Adjusted Odds Ratio [AOR]: 1.8, 95% Confidence Interval [CI]: 1.3–2.4). Similarly, FSWs who were not registered with an NGO/sex worker collective were 40% more likely to report non-disclosure of violence against those registered (58% vs. 53%, AOR: 1.4, 95% CI: 1.1–1.9).

**Conclusions:**

Non-disclosure of physical violence is quite high among FSWs which can be a barrier to the success of violence reduction efforts. Immediate efforts are required to understand the reasons behind non-disclosure based on which interventions can be developed. Community collectivisation and designing gender-based interventions with the involvement of non-paying partners should be the way forward.

## Introduction

Female sex workers (FSWs) are recognized as the most vulnerable population group to HIV infection.[1] Empirical evidence suggests that they are at a greater risk of experiencing violence, contracting sexually transmitted infections (STIs), including HIV and stigmatisation.[2], [3] Research reports suggest violence against FSWs can increase their vulnerability to HIV risk through several mechanisms.[4], [5] Studies in India report that about 10%–50% of FSWs experienced physical violence at the time of the survey.[2], [6]–[10] Paying partners, police, brokers, madams and non-paying partners are found to be the main perpetrators of violence whereas the perpetration from strangers or fellow FSWs were less prevalent. Sex workers generally considered violence as a part of their job and they lack proper information about their rights.[1] Many FSWs do not disclose or report their experience of violence because of fear of negative repercussions and consequences of disclosure. Moreover, previous research reports suggest that disclosure of violence is not an individual decision of an FSW but depends on the enabling societal contexts [11], [12].

In India, up-scaled HIV prevention programmes have been implemented since early 2000.[13], [14] Beside the components of STI prevention and treatment, these programmes also worked to reduce violence among FSWs.[15] As part of this strategy, 24-hour crisis response teams were established in each area comprising staff from programme implementing non-governmental organisations (NGOs), FSWs and human rights lawyers to sensitise perpetrators of violence and address the incidence of violence as and when they were reported by FSWs.[2], [16] Programme monitoring data suggest that these crisis response teams were able to address the majority of violence cases within 24 hours of reporting.[16] Data from multiple rounds of surveys also indicate that the incidence of violence has reduced among FSWs over time.[2] However, studies have not assessed whether the rate of reporting of violence has increased over time. One of these studies assumed that reporting of violence cases improved after the establishment of crisis response team, but they did not provide any scientific data to validate this assumption. [16] More specifically, no studies have attempted to understand the pattern of disclosure of violence among FSWs, that is, which kind of violence is being reported and which is not reported. This is critical because unless FSWs report the experience of violence, it cannot be redressed through any mechanism. Therefore, this study aims to study the rate of non-disclosure of violence among FSWs in India by perpetrators of violence and exposure to HIV prevention programmes.

## Materials and Methods

### Data

Data were drawn from the Integrated Behavioural and Biological Assessment (IBBA), a cross-sectional survey conducted among 10,618 FSWs during March–December 2009–10 across four states (in 23 districts) of southern and western India: three from southern India (Andhra Pradesh, Karnataka and Tamil Nadu) and one from western India (Maharashtra). FSWs who were 18 years or older had sold sex in cash/kind in the one month prior to the survey were interviewed. This study is based on a sub-sample of FSWs who reported experience of physical violence in six months preceding the survey.

Respondents were selected using a two-stage probability based sampling method. For selection of FSWs soliciting in public places such as park, street corner, bus stand, time location cluster sampling was used. However, for the selection of brothel and home based FSWs, conventional cluster sampling was used. The overall survey design including district selection, sample size calculation and participant recruitment has been described in detail elsewhere.[17] Face-to-face interviews were conducted by trained field workers in the local language of the state, using a structured questionnaire that included questions on socio-demographic characteristics, sexual behaviour, mobility, experience of violence and programme exposure. Interviews were conducted in private locations so that respondents’ confidentiality can be ensured.

### Ethics Statement

Statutory approval for conducting the IBBA and its protocols was obtained from the Government of India’s health ministry screening committee. A comprehensive consent process was adopted: respondents were first informed in detail about all aspects of the survey, following which written consent was separately obtained for the behavioural and biological components.

### Measures

The key outcome measure in this study is non-disclosure of violence among FSWs. Non-disclosure of violence was assessed among FSWs who reported experience of physical violence in last six months. A question was asked to individuals experiencing violence on who did they tell about the experience before the interview with response categories: did not tell anyone, fellow FSW, friend/relative/family member who is not a sex worker, staff from NGO. A response of “did not tell anyone” was considered as non-disclosure of violence (coded as 1 for non-disclosure and 0 for disclosure to someone). Disclosure to either fellow FSW and/or staff from NGO was considered as disclosure to an NGO worker/peer. HIV prevention programs in India recruit FSWs from a locality (identified as peer educator) to provide HIV prevention services (such as condom distribution, behaviour change communication and crisis response) in the same locality. Hence, while responding to the question on individuals to whom FSWs disclosed there violence, there is a high likelihood that FSWs would have indicated peer educators as fellow FSWs rather than NGO worker. Therefore, responses on Fellow FSWs were included in the category of NGO worker/peer category. Disclosure to family member/friend/relative who is not a sex worker was defined as disclosure to friend/relative.

FSWs experiencing physical violence in the past six months were also asked about the frequency of violence experienced (recoded as once, 2–5 times and 6+ times) and perpetrators in those cases (Response categories included: stranger, pimp, madam/broker, fellow FSW, paying partner, non-paying regular partner and police). Non-paying partners were individuals who don’t pay any cash in exchange for sex from the sex workers and these types of partners include husbands, boyfriends and lovers. In this study, fellow FSW, pimp and madam/broker were combined and represented as broker/fellow FSW in the analysis. Similarly, paying partner and stranger was combined in the analysis. These groups were done to ensure sufficient cell frequencies in each category without distorting the nature of grouping. Exposure to HIV prevention programmes was assessed by asking whether FSWs were registered with NGOs implementing HIV prevention programme in the district or a member of a sex worker collective in the district.

Information on socio-demographic variables like age (continuous), marital status (currently married, never married and formerly married), education (no formal education, formal education), sources of income (only from sex work and income from other sources beside sex work), alcohol consumption in past one month (categorised as no and yes), place of solicitation (grouped into home based, brothel based and street based) and duration in sex work (continuous) were assessed using single item questions. These variables were used as covariates in the regression analyses while examining the effect of degree and perpetrators of violence on non-disclosure of violence.

### Statistical Analyses

Univariate, bivariate and multivariate analyses were performed. Univariate analysis was conducted to present profile of the FSWs and disclosure rates of violence experience. Bivariate analysis was used to present the prevalence of non-disclosure of violence by programme exposure, degree of violence and perpetrators of violence. The strength of association of these predictor variables with outcome measure was measured using the Chi-square test. Multilevel multiple logistic regression models were used to examine predictors of non-disclosure of violence where individuals were nested within survey districts. Results were presented in the form of percentages (unadjusted), adjusted odds ratios (AOR) and their corresponding 95% confidence interval (CI). Additionally, intra-class correlation coefficients (ICC) and median odds ratio (MOR) were presented to infer the district level variation. All analyses were carried out using STATA version 12.1.

#### Methodological consideration

Table 1 indicates a considerable overlap between perpetrators of violence. In such a scenario, it is difficult to examine the effect of perpetrators of violence on non-disclosure of violence in a multivariate analysis. Therefore, respondents who reported more than one perpetrator of violence irrespective of their disclosure status were excluded from the analysis. Preliminary data analysis suggests that around 82% of the respondents reported only one perpetrator. This resulted in an analytical sample of 1341 FSWs, though 1631 FSWs had experienced violence in the past six months. Of the 290 observations that were excluded, there were 191 cases with a stranger/paying partner, 140 cases with police, 89 cases with broker/FSWs and 78 cases with paying partner as perpetrators of violence.

**Table 1:** Perpetrators of violence by disclosure of violence among female sex workers who experienced physical violence in the past six months, India, N = 1631.

Perpetrators ofviolence	Disclosed experience ofviolence to someone(N = 788)	Did not disclose experience ofviolence to someone (N = 843)
Paying partner/stranger	58.3	54.6
Police	20.3	16.3
Non-paying partner	17.9	25.2
Broker/FSW	25.4	12.8

## Results

The socio-demographic profile of FSWs who experienced violence in the past six months has been presented in Table 2. FSWs were, on average, 30 years old (standard deviation [SD]: 7 years) and practicing sex work for about six years (SD: 5 years). More than three-fifths were having no formal education (61%), and currently married (63%). About three-fifths of the FSWs were soliciting clients at street based settings (57%) and had income only from sex work (58%). About three-quarters (74%) were registered with an NGO/sex worker collective. More than half (57%) of FSWs experienced violence only once in the past six months and a little more than one-third (37%) experienced violence 2–5 times in the past six months. Paying partners/strangers were mentioned as the main perpetrators of violence (54%) followed by non-paying partners (21%), broker/fellow FSWs (14%) and police (12%).

**Table 2:** Profile of female sex workers who experienced physical violence in the past six months, India, N = 1341.

Background characteristics	% age or mean (SD)$ (N = 1341)
Age, mean (SD)$	30.5 (7.2)
No formal education	60.7
Currently married	63.0
Had income only from sex work	58.1
Duration in sex work, mean (SD)$	6.3 (5.5)
Consumed alcohol in past month	60.4
**Place of solicitation**	
Home based	18.3
Brothel based	24.9
Street based	56.8
Registered with NGO/sex worker collective	73.8
**State**	
Andhra Pradesh	41.1
Maharashtra	30.6
Tamil Nadu	14.8
Karnataka	13.7
***Violence related characteristics***	
**Frequency of experiencing violence**	
Once	56.5
2–5 times	37.1
6+ times	6.3
**Perpetrator of violence**	
Paying partner/stranger	54.3
Police	11.7
Non-paying partner	20.5
Broker/FSW	13.5

$ SD: Standard Deviation.

**Figure 1:**
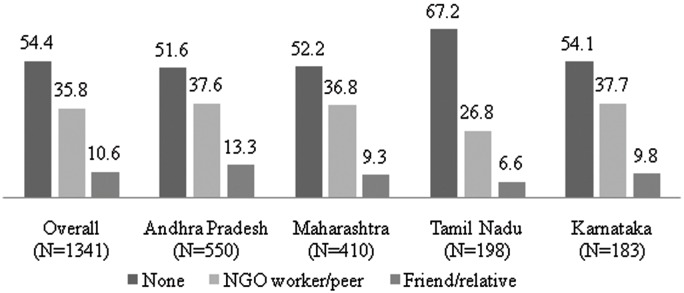
Rate of disclosure of experience of physical violence in six months prior to survey among female sex workers in India, N = 1341.

Out of the 1341 FSWs who experienced violence in the past six months, a little more than half (54%) did not disclose the incident to anyone, around one-third (36%) disclosed to an individual working with NGO worker/peer (Figure 1). The rate of non-disclosure was higher among FSWs in Tamil Nadu (67%) followed by Karnataka (54%), Maharashtra and Andhra Pradesh (52%). The rate of disclosure to NGO worker/peer was lowest in Tamil Nadu (27%) followed by Maharashtra (37%), Karnataka and Andhra Pradesh (38%).

The findings of multilevel analysis have been presented in Table 3. FSWs who experienced violence from non-paying partners were more likely not to disclose the experience to anyone as compared to those experienced violence at the hands of paying partners/strangers (68% vs. 53%, AOR: 1.8, 95% CI: 1.3–2.4). Similarly, non-disclosure is 67% more likely if the perpetrator is paying partner/stranger as against broker/fellow FSW (53% vs. 41%, AOR: 0.6, 95% CI: 0.4–0.9). FSWs who were not registered with NGO/sex worker collective were 40% more likely not to disclose experience of violence as compared to those who are registered (58% vs. 53%, AOR: 1.4, 95% CI: 1.1–1.9). Multilevel model estimated that the proportion of the variance in non-disclosure of violence between districts is about 7% (ICC: 0.072). Moreover, if a sex worker who experienced violence moved to another district with a higher probability of non-disclosure, the median increase in their odds of non-disclosure would be 1.6-fold (MOR: 1.6).

**Table 3:** Unadjusted rate of non-disclosure of violence and adjusted odd ratio with corresponding 95% confidence interval predicting the odds of non-disclosure with their degree of violence, perpetrators of violence and programme exposure as predictor variables in India (N = 1341).

Characteristics	%ge (n/N)	AOR (95% CI)^1^
**Frequency of experiencing violence**		
Once	53.8 (408/758)	Referent
2–5 times	54.0 (269/498)	1.0 (0.8–1.3)
6+ times	62.4 (53/85)	1.3 (0.8–2.2)
**Perpetrator of violence**		
Paying partner/stranger	53.0 (386/728)	Referent
Police	52.9 (83/157)	1.1 (0.7–1.6)
Non-paying partner	68.0 (187/275)	**1.8 (1.3–2.4)**
Broker/fellow FSW	40.9 (74/181)	**0.6 (0.4–0.9)**
**Registered with NGO/sex worker collective**		
No	58.2 (205/352)	**1.4 (1.1–1.9)**
Yes	53.1 (525/989)	Referent
**State**		
Andhra Pradesh	51.6 (284/550)	Referent
Maharashtra	52.2 (214/410)	0.9 (0.5–1.6)
Tamil Nadu	67.2 (133/198)	**2.0 (1.1–3.7)**
Karnataka	54.1 (99/183)	1.2 (0.6–2.2)
*Intra-class correlation*	0.072	
*Median Odds Ratio*	1.62	

1 AOR: Adjusted Odds Ratio, CI: Confidence Interval.

Multilevel models were adjusted for age, education, marital status, source of income beside sex work, duration in sex work, place of solicitation, and alcohol consumption.

## Discussion

Research reports in the last decade have demonstrated that FSWs who experience violence are more likely to report inconsistent condom use and inability to negotiate for condom use with sexual partners. [1], [2], [18]–[20] Researchers have attributed this to the experience of violence where FSWs would have chosen their own physical security over safe sex practice.[18], [21] In addition, post-hoc analysis from this study suggests that HIV prevalence among FSWs who experienced violence in past six months is 17% (15% among those did not disclose versus 19% among those who disclosed). Therefore, addressing violence among sex workers is important to the success of HIV prevention programmes. However, violence redresssal depends on the extent of reporting of the violence incidence by FSWs. In India, violence related issues, particularly association with FSWs’ HIV risk, have been studied extensively with limited attention to the disclosure of violence. This study is one of the first to examine rates of non-disclosure of physical violence and its association with the degree of violence, perpetrators of violence and programme exposure among FSWs in India. The study found that more than half of the FSWs who experienced violence did not disclose the incident to anyone and only two-fifths share the experience with an NGO worker or peer. Moreover, multilevel analysis indicated a considerable amount inter-district variation in non-disclosure of violence. Violence perpetrated by non-paying partners were more likely to be undisclosed than violence perpetrated by paying partners/strangers; more than two-thirds violence perpetrated by non-paying partners only were not disclosed by FSWs it to anyone. More importantly, the non-disclosure of violence was significantly higher among FSWs who were not registered with an NGO/sex worker collective than those registered indicating the contribution of HIV prevention programmes working towards violence reduction.

The study found one in every two FSWs who experienced violence did not disclose the violence experience to anyone. One of the reasons for such high rate of non-disclosure could be that FSWs did not perceive the degree of violence too severe and hence, may not have reported to anyone. Moreover, this can be the reason that non-disclosure was not associated with the degree of violence where even after repeated experience of violence, FSWs did not report it. There can be two more reasons for high rates of non-disclosure. First of these could be lack of enabling environment or a support system to which FSWs can share the experience. Second is related to awareness of sex workers about their legal rights. Earlier research in India has shown that HIV prevention programmes have made sincere efforts to improve both enabling environment and awareness about their legal rights.[2], [16], [22] However, the findings from this study highlight some important questions which are relevant both to programme implementers and researchers. To what extent FSWs are aware of their legal rights? Even if they are aware, why disclosure is not happening? Is it that still some of the violence is not considered as violence by FSWs? In-depth research is required to find answers to these questions. Study findings suggest that FSWs who were part of an NGO/sex worker collective were more likely to disclose. Evidence from previous research suggest that FSWs registered with NGO/sex workers collectives are more aware about their legal rights and different services are assessed by them with support of NGOs or some other HIV programs.[23] However, even a considerable proportion of FSWs who were part of the NGO/sex worker collective suggests that more effort is required to increase disclosure of violence experience. Disclosure of violence has to be improved because without disclosure, violence-related issues cannot be addressed and hence, vulnerability of FSWs cannot be reduced.

The study demonstrated that non-disclosure of violence is considerably higher if the perpetrator is a non-paying partner compared to any other type of perpetrators. In addition, compared to FSWs not registered with an NGO/sex worker collective, those registered were more likely to disclose the experience of violence. These two findings highlight the success of HIV prevention programmes working towards reducing violence from clients, police and brokers/pimps/brothel madams. HIV prevention programmes in India have worked over the years to sensitize different stakeholders on the violence issues. In addition, they have provided legal assistance to FSWs to deal with violence from clients/strangers or police.[16] This would have resulted in better reporting of violence to NGOs; moreover, post-hoc analysis also suggests that half of the violence perpetrated by paying partner/stranger, police or broker/fellow FSW were reported to NGO worker/peer.

The non-disclosure of violence perpetrated by non-paying partner in this study can be compared with the intimate partner violence faced by women in the general population. Data from a large scale demographic health survey also indicated that around 21% currently married women experienced physical abuse from their intimate partners in the last 12 months.[24] Interestingly, the present study also suggests a similar proportion of FSWs who experienced violence were perpetrated by non-paying partners. Drawing evidence from the general population, the high rate of non-disclosure of non-paying partner perpetrated violence can be attributed to the prevalent male-dominance in Indian societies where gender norms and attitudes support intimate partner violence. A research report from India suggests that a large proportion of women (96%) from general population believe that intimate partner violence is acceptable in at least one circumstance.[24] As discussed earlier, the other reason for low level of disclosure of violence can be lack of knowledge among individuals on the existing legal rights and provisions in the judiciary system. One can also argue that severity of violence perpetrated by non-paying partners is lesser than violence perpetrated by other partners and hence, the rate of disclosure is less when violence perpetrated by the former. However, post-hoc of analysis of the data suggests that the frequency of being physically abused (considered as a proxy for severity of violence) was more when perpetrator was non-paying partner than any other type of perpetrator. In the past six months, 58% of FSWs reported being beaten at least two times by non-paying partners against 40% being beaten two times or more by other type of perpetrators. Therefore, the severity of violence may not be an underlying factor for not disclosing experience of violence among this group of sex workers in India. A study conducted in five states of India also noted that even though intimate partner violence was widespread, only few women sought some sort of help from either from peer support group or women’s group or local government.[25] Therefore, there is a need to increase awareness among FSWs to recognise physical abuse from non-paying partners as violence and ability to show objection to any such violence. A study among FSWs in India noted that those who came in contact of local NGOs got relief from experience of violence which is also supported the findings of this study [23]. Though the incidence of violence from clients or police has reduced and reporting has improved over time due to the advocacy efforts of HIV prevention programmes, a lot still needs to be done to improve the reporting of non-paying partner violence.

The findings of the present study should be examined in light of certain limitations. First, responses to violence victimization, non-disclosure and about the perpetrators of violence are based on self-reports and there may be under reporting. Second, the question on the perpetrator of violence was a question with multiple responses possible. However, in only 18% of the cases multiple perpetrators were reported, and therefore there is no reason to believe the study findings will not hold true if the excluded cases were included in the analysis. Moreover, post-hoc analysis suggests that even after including these 18% of cases as a separate category (as multiple perpetrators), the results hold true. Third, the study did not collect data on the severity and form of violence as well as on the reason of violence and measures taken after the experience of violence. Data on these aspects would have helped better to explain the nature and context of violence experience. We recommend that future research in this direction should collect data on these aspects.

Despite these study limitations, the study findings have important policy implications both at the micro and macro level of HIV prevention programmes. At the micro level, advocacy efforts till date have mainly focussed on sensitization of police and other stakeholders of sex work and a very little on sexual partners. However, we recommend that efforts should also be to involve non-paying partners as well as clients of sex workers as part of advocacy effort. Particularly for non-paying partners, interventions focussed on gender equity measures may be implemented. Moreover, sensitization on gender norms, particularly on patriarchal attitudes non-paying partners should be addressed in these counselling sessions. Lack of social security, isolation from the crisis response system and fear of retaliation may prevent FSWs from disclosing experience of violence. Therefore, enabling environment and peer support are two other important aspects that can improve the disclosure. Therefore, effort to improve enabling environment should continue. The other approach to reduce violence and improve disclosure is bringing FSWs together through community collectivization. Research from India has already demonstrated that community collectivization improves self-efficacy of FSWs which can in turn help them to raise their voice against violence.[22], [26]–[28] Moreover, a case study from south India has demonstrated that collectivization of FSWs improved their self-efficacy for crisis response and improved enabling environment.[22] Efforts on counselling, and sensitization on violence related issues should continue. Activities to increase awareness on FSWs’ basic human rights should be organised. In conclusion, disclosure of violence is critical to success of violence reduction programmes among FSWs. Therefore, immediate efforts are required to understand the reasons behind non-disclosure based on which interventions can be developed.
